# Sangerbox: A comprehensive, interaction‐friendly clinical bioinformatics analysis platform

**DOI:** 10.1002/imt2.36

**Published:** 2022-07-08

**Authors:** Weitao Shen, Ziguang Song, Xiao Zhong, Mei Huang, Danting Shen, Pingping Gao, Xiaoqian Qian, Mengmeng Wang, Xiubin He, Tonglian Wang, Shuang Li, Xiang Song

**Affiliations:** ^1^ Bioinformatics R&D Department Hangzhou Mugu Technology Co., Ltd Hangzhou China; ^2^ Department of Cardiovascular Medicine Shanghai University of Medicine & Health Sciences Affiliated Zhoupu Hospital Shanghai China; ^3^ Cardiovascular Center The Fourth Affiliated Hospital of Harbin Medical University Harbin Heilongjiang China; ^4^ College of Basic Medical Harbin Medical University Harbin Heilongjiang China; ^5^ Renal Division, Department of Internal Medicine Xinhua Hospital Affiliated to Shanghai Jiao Tong University of Medicine Shanghai China; ^6^ Oncology Research Center Beidahuang Industry Group General Hospital Harbin Heilongjiang China

## Abstract

In recent decades, with the continuous development of high‐throughput sequencing technology, data volume in medical research has increased, at the same time, almost all clinical researchers have their own independent omics data, which provided a better condition for data mining and a deeper understanding of gene functions. However, for these large amounts of data, many common and cutting‐edge effective bioinformatics research methods still cannot be widely used. This has encouraged the establishment of many analytical platforms, a portion of databases or platforms were designed to solve the special analysis needs of users, for instance, MG RAST, IMG/M, Qiita, BIGSdb, and TRAPR were developed for specific omics research, and some databases or servers provide solutions for special problems solutions. Metascape was designed to only provide functional annotations of genes as well as function enrichment analysis; BioNumerics and RidomSeqSphere+ perform multilocus sequence typing; CARD provides only antimicrobial resistance annotations. Additionally, some web services are outdated, and inefficient interaction often fails to meet the needs of researchers, such as our previous versions of the platform. Therefore, the demand to complete massive data processing tasks urgently requires a comprehensive bioinformatics analysis platform. Hence, we have developed a website platform, Sangerbox 3.0 (http://vip.sangerbox.com/), a web‐based tool platform. On a user‐friendly interface that also supports differential analysis, the platform provides interactive customizable analysis tools, including various kinds of correlation analyses, pathway enrichment analysis, weighted correlation network analysis, and other common tools and functions, users only need to upload their own corresponding data into Sangerbox 3.0, select required parameters, submit, and wait for the results after the task has been completed. We have also established a new interactive plotting system that allows users to adjust the parameters in the image; moreover, optimized plotting performance enables users to adjust large‐capacity vector maps on the web site. At the same time, we have integrated GEO, TCGA, ICGC, and other databases and processed data in batches, greatly reducing the difficulty to obtain data and improving the efficiency of bioimformatics study for users. Finally, we also provide users with rich sources of bioinformatics analysis courses, offering a platform for researchers to share and exchange knowledge.

## Graphical abstract

Sangerbox with a user‐friendly interface supports differential analysis, correlation analyses, pathway enrichment analysis, weighted correlation network analysis, and so on. A new interactive plotting system that allows users to adjust the parameters in the image. It has organized GEO, TCGA, ICGC, and other databases; a rapid batch processing reduces the difficulty in data acquirement, greatly improving the efficiency.

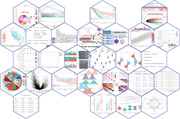

## SUMMARY

In recent decades, the continuous development of high‐throughput sequencing technology has increased data volume in medical research [1]. At the same time, almost all clinical researchers have their own independent omics data, which provided a better condition for data mining and a deeper understanding of gene functions. However, due to the larger amount of data, many common and cutting‐edge effective bioinformatics research methods still cannot be widely used. This has encouraged the establishment of many analysis platforms and databases to accommodate the demands of users, for instance, Qiita for amplicon data and analysis [2], ImageGP for plotting [3], QIIME for microbiome analysis and visualization, and Majorbio cloud for multiomics [4], have been developed for omics research. Some databases or servers provide solutions for special problems. Metascape [5] was designed to provide functional annotations of genes and function enrichment analysis; metaOrigin [6] supports metabolome original analysis from microbiome; Gene2vec for m6A prediction [7]; iNAP for network analysis [8]; PsRobot [9] for small RNA meta‐analysis; DeepKla [10] for protein lysine lactylation site prediction. Additionally, some web services are outdated, and inefficient interaction often fails to meet the current needs of researchers. Therefore, the demand to complete massive data processing tasks urgently requires a comprehensive bioinformatics analysis platform.

Hence, we have developed a website platform, Sangerbox (http://vip.sangerbox.com/). The platform as a user‐friendly interface supports differential analysis and provides interactive customizable analysis tools, including various kinds of correlation analyses, pathway enrichment analysis, weighted correlation network analysis (WGCNA) [11] as well as some other common tools and functions. Users only need to upload their own corresponding data into Sangerbox, select required parameters, submit, and collect the results after the task has been completed. We have also established a new interactive plotting system that allows users to adjust the parameters in the image; moreover, optimized plotting performance enables users to adjust large‐capacity vector maps on the website. At the same time, Sangerbox has integrated GEO, TCGA, ICGC and other databases and processed the data in batches, which greatly reduces the difficulty to obtain data and improves the efficiency of bioinformatics analysis for users. Additionally, we also provide users with rich sources of bioinformatics analysis courses, creating a platform for researchers to share and exchange knowledge.

**Figure 1:**
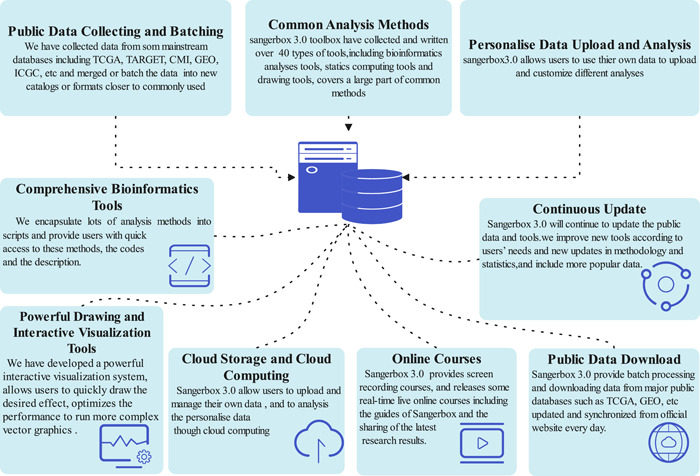
Content and framework of Sangerbox

Since August 2021 when the Sangerbox cloud platform was established, it has accumulated more than 20,000 users, running 150,428 times for analysis, plotting, downloads, and other tasks, and previous versions of Sangerbox have been used 813,816 times. This proves that a comprehensive, interaction‐friendly bioinformatics data analysis platform is greatly needed and welcomed by researchers in the field. The content and framework of Sangerbox are shown in Figure 1. Such a platform can greatly facilitate data mining, scientific discussion, and treatment discovery processes in a wide range of biological and clinical research areas.

### Convenient, powerful and interactive analyzing and plotting tools

**Figure 2:**
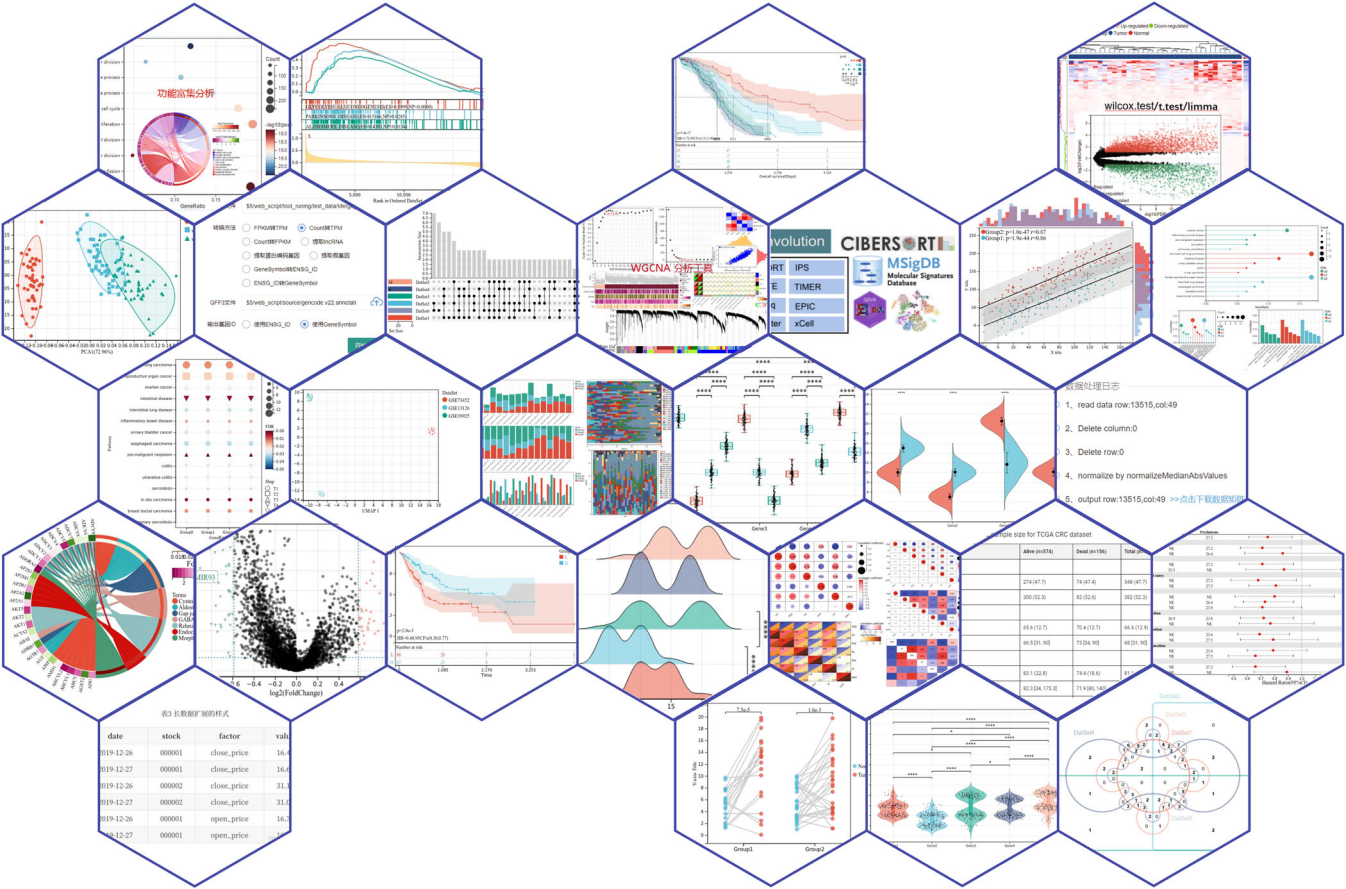
Example of toolbox

The Sangerbox platform accelerates the analysis of researchers' data, improves the utilization of both public and personal data, and contributes to the development of clinical research. Bioinformatics analysis has long been difficult for clinical and specialized experimental researchers to get started because whether it is code programming skills, mathematical skills, or statistical knowledge, which all need to be built up over time, often require great effort from researchers if starting from scratch. In addition, many analysis processes demand high‐performance computer services. Here, the Sangerbox platform with a greater amount of data and calculations has higher performance than personal computers in completing different tasks. With only basic knowledge principles and analytical purposes of the tools, over 30 different types of tools in the Sangerbox platform can more efficiently help researchers complete their analysis, simplifying the learning process and reducing learning costs while speeding up the data processing process and digesting the growing amount of bioinformatics data (Figure 2). Meanwhile, the use of the cloud platform model allows researchers to easily break through the limits of computing power and analysis memory, enabling the application of complex algorithms and mining of larger data. The platform also provides a visual web interface on which researchers could use plotting tools and bioinformatics analysis tools by entering their own biological data and setting parameters. At present, the Sangerbox platform integrates more than 100 commonly used calculation and analysis methods, and around 40 kinds of tools provided roughly cover statistical, analysis, and visualization tools.

The plotting tools cover many common chart types, such as forest map, radar map, heat map [12], violin plots, box plot [13], Venn diagram [14], and circlize plot [15] that provides statistical analysis functions while plotting. These can reflect statistical information and allow users to test parameters or nonparameters within the group during data plotting. Second, the data input format has been adjusted on Sangerbox to be more in line with common data, making the daily process of plotting icons more convenient. Moreover, our plotting system is more suitable for use on web pages. Compared with commonly used ggplot2 [16], ComplexHeatmap [12], or other R packages [17], the Sangerbox platform is intuitive and easy to operate. At the same time, we have also optimized the performance and can support various device performances.

Furthermore, the Sangerbox platform also provides users with a large number of widely used bioinformatics analysis tools covering text processing and data standard preprocessing (standardization, normalization, etc.). Data analysis includes dimension reduction, clustering, difference analysis, and so on. In addition, some common bioanalysis processes, such as weighted correlation network analysis (WGCNA) [11], principal component analysis (PCA) [18], survival analysis [19], Gene Set Enrichment Analysis (GSEA) [20], and Limma differential expression analysis [21], are accommodated as well.

Sangerbox will continue to improve new tools according to users' needs and new updates in methodology and statistics so that researchers using the Sangerbox platform can reduce the learning cost and more clinical bioinformatics data can be more efficiently processed, thereby contributing to the development of clinical research.

### Powerful interactive visualization interface

We have developed a new visual interaction system with the goal of “what you see is what you get” and without cumbersome programming code or complex parameter settings. The platform is established based on d3.js and jquery.js. The interactive visual interface, which was designed using JavaScript, allows users to intuitively obtain vector graphics using mouse clicks and input to set parameters in web pages, thereby realizing our goal of “what you see is what you get.” Furthermore, Sangerbox also supports users to export bitmap or vector graphics in different formats to further support their research.

In addition, it is well acknowledged that the vector plotting adjustment process consumes great arithmetic power. Thus, we have optimized the plotting performance of vectors on the web site, allowing researchers in different working environments to adjust their vector images according to their own needs.

**Figure 3:**
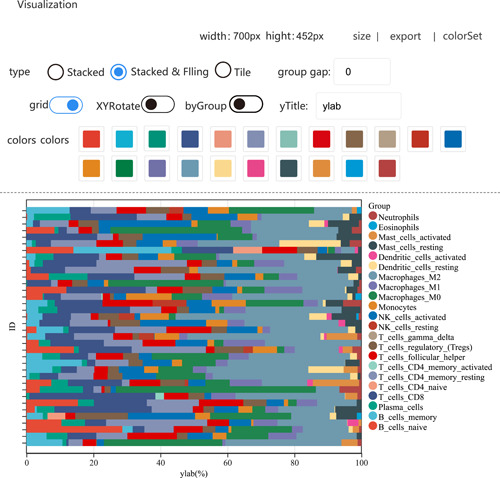
The example of the histogram visualization panel.

As shown in Figure 3, a stacked histogram was displayed as an example. Sangerbox supports data input to obtain initial pictures of histogram form, group spacing, grid switch, and so on, and then users could select parameters by mouse click or direct input. The same interface is also provided for plotting charts on the web page.

### Public data download and processing

The Sangerbox platform supports easier download of public data and simple but fast data preprocessing, which helps researchers to obtain and apply data more quickly. These data are derived from The Cancer Genome Atlas (TCGA) [22], International Cancer Genome Consortium (ICGC) [23], Gene Expression Omnibus (GEO) [24], and other databases with clinical follow‐up information, clinical data, mutation data, and expression profile data. Sangerbox also provides preprocessing functions for the gene expression profile matrix in NCBI's GEO database, supporting a direct use for reannotation, standardization, and other steps. At the same time, a new category for TCGA, ICGC, TARGET (therapeutically applicable research to generate effective treatments) and other databases have been integrated to better accommodate the using habits of general researchers and help users obtain public data more simply and quickly.

The Sangerbox platform has built a complete course sharing platform. While providing screen recording courses, we also release some real‐time live online courses. This can not only help scientific research users get familiar with how to use the platform but also informs them about the cutting‐edge research trends and research methods in certain fields.

### Users and publications

From August 2021, Sangerbox has accumulated more than 20,000 scientific research users during about half a year and has completed 150,428 data analyses, mining, and mapping tasks. In the last few years, over 300 documented journal articles have cited the use of Sangerbox in their methodology. The platform will constantly upgrade and update the content.

### Methods and techniques

We employed the SpringCloud framework, java 11,10.2.30‐MariaDB to build the website, website backstage, and database. Web pages were constructed using the layui framework, html 5, and JavaScript languages. R 3.6.3, R 4.0.1, Perl, and JavaScript languages were applied to write and convert into arithmetic and analysis scripts.

## AUTHOR CONTRIBUTIONS

Xiang Song and Shuang Li conceived the idea of developing the Sangerbox platform. Weitao Shen completed the technical construction of the platform and the realization of each function and wrote the manuscript. Ziguang Song and Xiao Zhong are responsible for collecting and collating data. Mei Huang was responsible for editing and revising the manuscript. Danting Shen, Xiaoqian Qian, Pingping Gao, Mengmeng Wang, Xiubin He, and Tonglian Wang completed the collection and arrangement of methods and materials for the platform. All authors contributed to the development of Sangerbox.

## CONFLICTS OF INTEREST

Weitao Shen is the developer of the platform. Xiang Song and Shuang Li are cofounders of the project. Ziguang Song, Xiao Zhong, Danting Shen, Xiaoqian Qian, Pingping Gao, Mengmeng Wang, Mei Huang, Xiubin He, and Tonglian Wang are employees and researchers of Hangzhou Mugu Technology Co., Ltd.

## Data Availability

We will view or use the personal uploaded data of the users with the corresponding permission of the users. The public data we collected and batched is available online. Supplementary materials (figures, tables, scripts, graphical abstract, slides, videos, Chinese translated version, and update materials) may be found in the online DOI or iMeta Science http://www.imeta.science/.
